# Bidirectional causal relationship between hypercholesterolemia and ischemic heart disease: a Mendelian randomization study

**DOI:** 10.3389/fcvm.2023.1302282

**Published:** 2023-12-07

**Authors:** Ying Jiang, Wenpeng Yu, Jianliang Zhou, Xiao Dong

**Affiliations:** ^1^ Department of Cardiovascular Surgery, The Second Affiliated Hospital of Nanchang University, Nanchang, China; ^2^ Department of Cardiovascular Surgery, Zhongnan Hospital of Wuhan University, Wuhan, China

**Keywords:** hypercholesterolemia, ischemic heart disease, Mendelian randomization, bidirectional causal relationship, genetic co-localization analysis

## Abstract

**Background:**

Ischemic Heart Disease (IHD) is a leading cause of morbidity and mortality worldwide. Hypercholesterolaemia, a metabolic syndrome distinguished by elevated cholesterol levels, is positively correlated with IHD, yet the precise causal relationship between these two health conditions remains to be clearly defined.

**Methods:**

We conducted a two-sample MR analysis using genetic variants associated with hypercholesterolemia and IHD. Various statistical techniques including MR-Egger, Weighted Median, Inverse Variance Weighted (IVW), Simple Mode, and Weighted Mode were employed. We also performed sensitivity analyses to assess pleiotropy, heterogeneity, and influence of individual SNPs. Furthermore, genetic co-localization analysis was performed to identify shared genes between hypercholesterolemia and IHD.

**Results:**

Our MR study illuminated a bidirectional causal relationship between hypercholesterolaemia and ischaemic heart disease. Utilising the IVW with multiplicative random effects, upon considering IHD as the outcome, we identified an OR of 2.27 (95% CI: 1.91–2.70, *p* = 1.68 × 10^−20^). Conversely, when hypercholesterolaemia was viewed as the outcome, the OR detected was 1.80 (95% CI: 1.58–2.05, *p* = 2.79 × 10^−19^). These findings remained consistent across various MR methods and sensitivity analyses. Additionally, our research pinpointed four co-localised genes CELSR2, PCSK9, LPA, and APOE as integral candidates implicated in the pathogenesis of both conditions, thereby suggesting shared common genetic causal variants and offering potential targets for innovative therapeutic strategies.

**Conclusion:**

bidirectional MR studies reveal genetic evidence of a potential causal link between hypercholesterolaemia and IHD. Notably, these findings also lend credence to the less traditional hypothesis that IHD may instigate hypercholesterolaemia episodes. Moreover, co-localisation analyses intimate the presence of shared genetic causal variants, paving the way for the development of new therapeutic strategies.

## 1. Introduction

Ischemic Heart Disease (IHD) is a major cause of global mortality, claiming over 9 million lives annually. With approximately 126.5 million cases globally, its impact on public health is significant. In the United States, it presents both a substantial health challenge and an economic encumbrance, with yearly costs estimated in the hundreds of billions (1–3). IHD is principally defined by diminished myocardial perfusion, instigated by a complex interplay of pathophysiological factors within the coronary artery wall's intimal layer. These elements include endothelial dysfunction, inflammation, thrombogenesis, and the aftereffects of angiogenesis and calcification. A notable contributor to this pathological nexus is the presence of oxidized serum lipids, a hallmark of hypercholesterolemia (4–6). Elevated LDL cholesterol levels lead to hypercholesterolemia, endothelial dysfunction, and increased atherosclerosis, potentially hastening death in patients with severe ischemic heart disease (7–9).

The chief contributors to hypercholesterolemia include an excessive biosynthesis of cholesterol and/or inadequate clearance of it. It's noteworthy that hypercholesterolemia amplifies the risk for developing atherosclerosis and ischemic heart disease (10). Although hypercholesterolemia's role as a significant risk factor for IHD is widely recognized (11), the suggestion of a causal relationship flowing in the opposite direction—that is, IHD potentially instigating the onset of hypercholesterolemia—presents a less conventional perspective. This concept implies a bidirectional causality between hypercholesterolemia and IHD, a process that warrants meticulous exploration. In our quest to decipher the causal link between the two, we employed an approach known as Mendelian randomization (MR). MR leverages genetic variants as instrumental variables tied to an exposure, consequently amplifying the dependability of the causal inference connecting an exposure and its subsequent outcome. This method helps to address potential challenges such as confounding factors and reverse causality (12, 13). If there exists a causal relationship between the exposure and the outcome, genetic variations influencing the exposure will likely affect the outcome as well. MR is akin to a natural randomized controlled trial, being based on Mendel's second law. This law proposes that alleles from distinct genes are assorted independently during gametogenesis. Consequently, the underlying premise of Mendelian randomization analyses is that the inheritance of a particular trait should remain independent of the inheritance of any other traits (14).

Hence, in our study, we employed a bidirectional MR analysis utilizing genetic variants linked to hypercholesterolemia and IHD. Our statistical methodology encompassed MR-Egger, Weighted Median, IVW, Simple Mode, and Weighted Mode. Additionally, we conducted sensitivity analyses to evaluate the influences of pleiotropy, heterogeneity, and individual single nucleotide polymorphisms (SNPs). Ultimately, we carried out a genetic co-localization analysis to identify shared genes between hypercholesterolemia and IHD. Gaining an understanding of the bidirectional relationship between hypercholesterolemia and IHD holds instructive value in terms of risk monitoring for individuals with either disease and identifying colocalized genes as potential targets for therapeutic interventions.

## 2. Materials and methods

### 2.1. Study design

**Figure 1:**
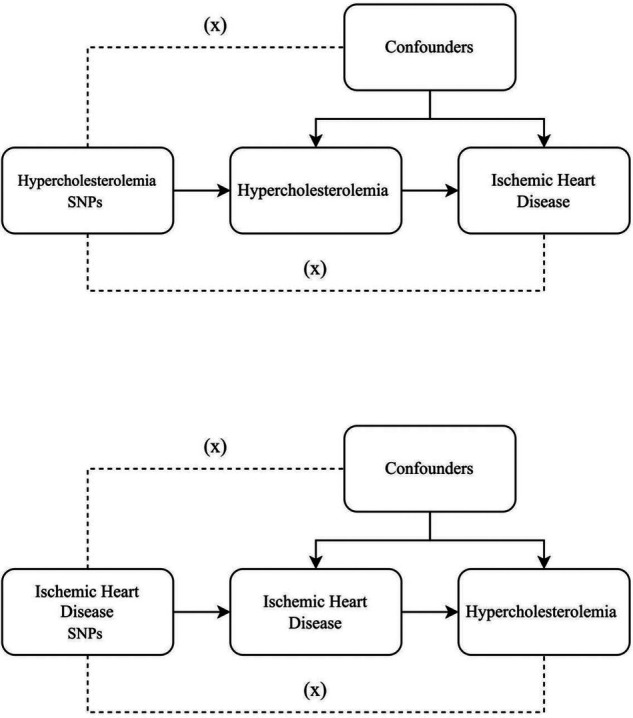
Three key assumptions underlying Mendelian randomization study design. The three different hypotheses are represented by three pathways. Hypothesis 1: SNP is associated with exposure. Hypothesis 2: SNP affects outcome only through exposure and not through any alternative causal pathways. Hypothesis 3: SNPs are completely independent of any potential confounding factors that influence exposure and outcome.

SNPs exemplifying genetic diversity were chosen as instrumental variables and underwent a two-sample MR analysis. Three core assumptions (15) were established as follows Figure 1: (1) a direct connection exists between instrumental variables and exposure; (2) instrumental variables remain unconnected to any potential confounders; (3) the impact of genetic variations on the results occurs solely through exposure. We use Mendelian randomization (MR) analysis to assess the two-way causal link between IHD and hypercholesterolemia. To further investigate the shared genetic basis between IHD and hypercholesterolemia, genetic colocalisation analysis was performed. This method allows us to assess whether the genetic signals identified in two independent association studies, such as those for IHD and hypercholesterolemia, are colocalized, indicating a common genetic mechanism underlying both traits. By integrating the results from the MR analysis with the colocalization analysis, we can gain a deeper understanding of the potential shared genetic influences and biological pathways involved in the development of IHD and hypercholesterolemia.

### 2.2. Genome-wide association analysis

Genome-wide association study (GWAS) databases (16), such as GWAS Catalog, IEU openGWAS, and NealELab, were explored and suitable datasets were obtained. Given that all data utilized was publicly available, no further ethical approval was necessary. To minimize the bias stemming from ethnicity-related confounding elements, the study's genetic background was limited to individuals of European descent. The IEU GWAS report encompasses two distinct traits: “Pure hypercholesterolemia” and “Ischaemic heart disease”. The first study, identified as ukb-b-12651, was conducted in 2018 by Ben Elsworth, while the second study, identified as ukb-d-I9_IHD, was carried out in the same year by Neale Lab. Both studies used phesant-derived variables from the UK Biobank and included participants of European descent, both males and females. The ukb-b-12651 study had a sample size of 463,010 participants, consisting of 22,622 cases and 440,388 controls. On the other hand, the ukb-d-I9_IHD study included a sample size of 361,194 participants, with 20,857 cases and 340,337 controls. Both studies employed the HG19/GRCh37 genome build and focused on binary GWAS categories, incorporating MR analysis. These reports are publicly accessible and were conducted as part of the MRC-IEU consortium.

### 2.3. SNP selection and statistical analysis

We adopted a two-sample MR approach, facilitated by the TwoSampleMR R package, to explore the two-way causal relationship between hypercholesterolemia and ischemic heart disease (16). MR analysis is performed by following a careful workflow to ensure the credibility and robustness of the results.

#### 2.3.1. Instrument selection

We first identified SNPs from the exposure dataset (hypercholesterolemia) and the outcome dataset (ischemic heart disease). SNPs were selected as IVs based on their genome-wide significance (*p*-value < 5 × 10 × 10^−8^) and linkage disequilibrium clumping with an *r*^2^ threshold of 0.001 and a 10,000 kb window to ensure their independence.

#### 2.3.2. Data harmonization

The summary-level data from GWAS for the selected SNPs were extracted, and the exposure and outcome datasets were harmonized to ensure the consistency of effect allele coding and alignment of SNP effect estimates (17). Any strand-ambiguous or palindromic SNPs were appropriately handled or excluded from the analysis.

#### 2.3.3. *F*-statistic calculation

We set a threshold of an *F*-statistic equal to or greater than 10 for conducting the MR analysis to reduce the potential impact of weak instrument bias (18). The *F*-statistic can be calculated using the formula: $F=\frac{R^{2}\cdot \left(N-2\right)}{1-R^{2}}$, where *N* is the sample size, *k* represents the number of instrumental variables (IVs), and *R*^2^ indicates the proportion of variation explained by the SNPs. The calculation of *R*^2^ for the 5 genome-wide significant SNP instrument was performed using the formula: $R^{2}=2\cdot \mathrm{EAF}\cdot (1-\mathrm{EAF})\cdot \beta^{2}$. In contrast, for the extended 10 SNP instrument, the calculation of *R*^2^ was conducted using the equation: $R^{2}=\frac{\beta^{2}}{\beta^{2}+N\cdot \mathrm{SE}{\left(\beta \right)}^{2}}$ (19). The calculation of *R*^2^ was determined based on the number of SNPs involved in the analysis. The equation includes variables such as EAF, beta, *N*, and SE (beta). EAF represents the effect allele frequency, beta is the estimated genetic effect on physical activity, *N* is the sample size of the GWAS for the SNP-physical activity association, and SE (beta) signifies the standard error of the genetic effect. By adhering to this threshold and utilizing the *F*-statistic, we can ensure that our MR analysis accounts for the strength of the genetic instruments and minimizes the risk of weak instrument bias, thus contributing to the validity of the causal inferences made from the analysis.

#### 2.3.4. MR analysis

We conducted the MR analysis using the harmonized data to estimate the causal effect of hypercholesterolemia on ischemic heart disease. Several MR methods, including the IVW, MR-Egger, and Weighted Median, were used to obtain robust causal effect estimates and account for potential biases in the analysis.

#### 2.3.5. Heterogeneity, pleiotropy, and sensitivity assessment

The Cochran's *Q* statistic was employed to assess the heterogeneity of individual SNP estimates. To assess the impact of individual SNPs on the overall causal estimate, a leave-one-out analysis was conducted. The MR-Egger regression intercept was examined for evidence of pleiotropy, and the funnel plot symmetry was visually inspected.

#### 2.3.6. Reverse direction analysis

We then reversed the exposure and outcome datasets by defining the exposure dataset for ischemic heart disease and the outcome dataset for hypercholesterolemia. Following the same procedure as described above, the MR analysis was performed to investigate the causal effect of ischemic heart disease on hypercholesterolemia.

#### 2.3.7. Result visualization

Scatter plots, forest plots, funnel plots, and leave-one-out plots were generated to visualize the results of both MR analyses, including the causal estimates, heterogeneity, pleiotropy, and sensitivity analyses. These visualizations allowed for a comprehensive assessment of the robustness and validity of the MR findings.

### 2.4. Colocalization analysis of Complex traits

We utilized the R package “ieugwasr” to obtain top hits from the UK Biobank dataset for two traits of interest (traits “ukb-b-12651” and “ukb-d-I9 IHD”). The top hits were sorted by their *p*-values. To perform the colocalization analysis, we first determined the best signals for each trait and created a range around these loci. We considered a region of ±90,000 base pairs (bp) for most of the top hits, with some exceptions where we adjusted the range based on the specific chromosomal position. The R package “coloc” was then employed to conduct a colocalization analysis between the two traits (20), using the “coloc.abf” function applied to the extracted and formatted data obtained from the “ieugwasr_to_coloc” function. After completing the colocalization analysis, we visualized the results using the “gassocplot” R package. The “stack_assoc_plot” function was applied to the output from the “coloc_to_gassocplot” function, generating a stacked Manhattan plot representing the genetic association signals from both traits.

## 3. Results

### 3.1. MR analysis of bidirectional associations between pure hypercholesterolemia and ischemic heart disease

In this study, we conducted a MR analysis to investigate the causal relationship between hypercholesterolemia and ischemic heart disease. We employed five MR methods, including MR Egger, Weighted Median, IVW, Simple Mode, and Weighted Mode, to obtain robust estimates of causal effects. All SNPs utilized in this study exhibited F statistics exceeding 10, indicating robust instrumental variables.

#### 3.1.1. Causal effect of hypercholesterolemia on IHD via forward MR

The main results for ischemic heart disease as the outcome are as follows: MR Egger: The odds ratio (OR) for the causal effect of hypercholesterolemia on ischemic heart disease was 2.95 (95% CI: 2.11–4.14, *p* = 1.09 × 10^−5^). Weighted Median: The OR for the causal effect was 1.87 (95% CI: 1.66–2.10, *p* = 6.25 × 10^−26^). Inverse Variance Weighted (IVW): The OR for the causal effect was 2.27 (95% CI: 1.91–2.70, *p* = 1.68 × 10^−20^). Simple Mode: The OR for the causal effect was 1.97 (95% CI: 1.58–2.47, *p* = 1.63 × 10^−5^). Weighted Mode: The OR for the causal effect was 1.91 (95% CI: 1.67–2.19, *p* = 3.87 × 10^−8^). We conducted several sensitivity analyses to evaluate the assumptions, pleiotropy, heterogeneity, and the influence of individual SNPs on our MR analysis. Pleiotropy: The MR-Egger regression test did not indicate significant pleiotropy (egger intercept = −1.66 × 10^−3^, SE = 9.49 × 10^−4^, *p* = 0.0995), suggesting that the genetic instruments are not biased due to horizontal pleiotropy. Heterogeneity: The Cochran's *Q* test for both MR-Egger (*Q* = 127.12, *df* = 16, *p* = 2.32 × 10^−19^) and Inverse Variance Weighted (IVW) (*Q* = 151.41, *df* = 17, *p* = 1.29 × 10^−23^) methods revealed significant heterogeneity across the genetic instruments. Due to the significant heterogeneity revealed by the Cochran's *Q* test in our MR-Egger and Inverse Variance Weighted (IVW) analyses, we opted to use the Inverse Variance Weighted method with multiplicative random effects in our study. Leave-one-out analysis: The analysis demonstrated that no individual SNP had a disproportionate influence on the overall causal estimates. The results remained consistent after excluding each SNP, suggesting the robustness of the MR findings. All of the above results can be found in Supplementary file 1.

**Figure 2:**
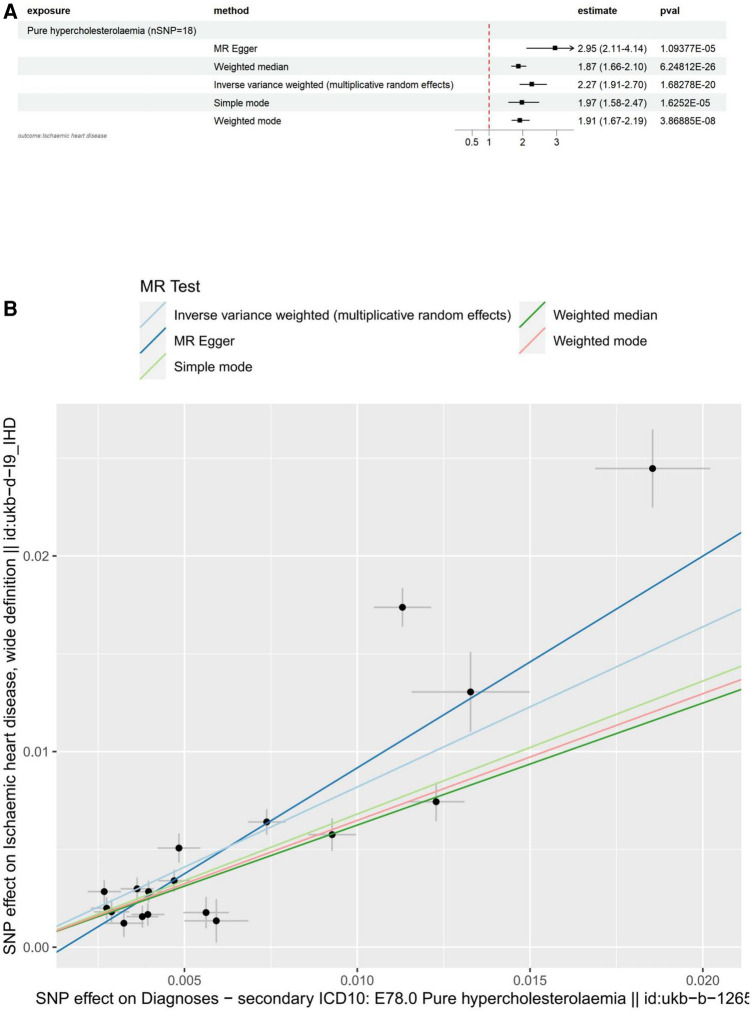
Visualization of hypercholesterolemia as an exposure factor in a two-sample MR. (**A**) Forest plots depicting the potential impact of SNPs associated with hypercholesterolaemia on IHD using five MR methods. (**B**) Scatter Plots Illustrating the Effect of SNPs on Hypercholesterolaemia and its Correlation with the Effect on IHD. Circles indicate marginal genetic associations with hypercholesterolemia and risk of IHD for each variant. Error bars indicate 95% CIs. IHD, ischemic heart disease; MR, mendelian randomization; SNP, single nucleotide polymorphism.

To visualize these results, we utilized forest plots (Figure 2A) and scatter plots (Figure 2B). While other visualization plots used to assess heterogeneity, polymorphism and sensitivity analysis include forest plots, funnel plots, and leave-one-out plots, which we can find in Supplementary Figure S1.

#### 3.1.2. Causal association of IHD with hypercholesterolemia via reverse MR

The main results for hypercholesterolemia as the outcome are as follows: MR Egger: The odds ratio (OR) for the causal effect of hypercholesterolemia on ischemic heart disease was 1.88 (95% CI: 1.43–2.48, *p* = 1.27 × 10^−4^). Weighted Median: The OR for the causal effect was 1.46 (95% CI: 1.34–1.58, *p* = 2.48 × 10^−20^). Inverse Variance Weighted (IVW): The OR for the causal effect was 1.80 (95% CI: 1.58–2.05, *p *= 2.79 × 10^−19^). Simple Mode: The OR for the causal effect was 1.44 (95% CI: 1.28–1.61, *p* = 1.01 × 10^−6^). Weighted Mode: The OR for the causal effect was 1.42 (95% CI: 1.30–1.55, *p* = 1.35 × 10^−8^).

Pleiotropy: The results of the MR-Egger regression analysis revealed no significant pleiotropy (egger intercept = −3.00 × 10^−4^, SE = 8.29 × 10^−4^, *p* = 0.720). This finding suggests that the genetic instruments used in our study are not biased due to horizontal pleiotropy, and the observed associations between hypercholesterolemia and ischemic heart disease are likely to reflect a causal relationship.

Heterogeneity: The Cochran's *Q* test for both MR-Egger (*Q* = 343.34, *df* = 27, *p *= 1.50 × 10^−56^) and Inverse Variance Weighted (IVW) (*Q* = 345.01, *df* = 28, *p* = 2.51 × 10^−56^) methods revealed significant heterogeneity across the genetic instruments. Due to the significant heterogeneity revealed by the Cochran's *Q* test in our MR-Egger and Inverse Variance Weighted (IVW) analyses, we opted to use the Inverse Variance Weighted method with multiplicative random effects in our study. This approach, which accounts for heterogeneity among the genetic instruments, provides a more accurate and robust estimation of the causal effect. Leave-one-out analysis: The analysis demonstrated that no individual SNP had a disproportionate influence on the overall causal estimates for the association between Ischaemic heart disease (wide definition) and pure hypercholesterolaemia. The results remained consistent after excluding each SNP, suggesting the robustness of the MR findings. All of the above results can be found in Supplementary file 2.

**Figure 3:**
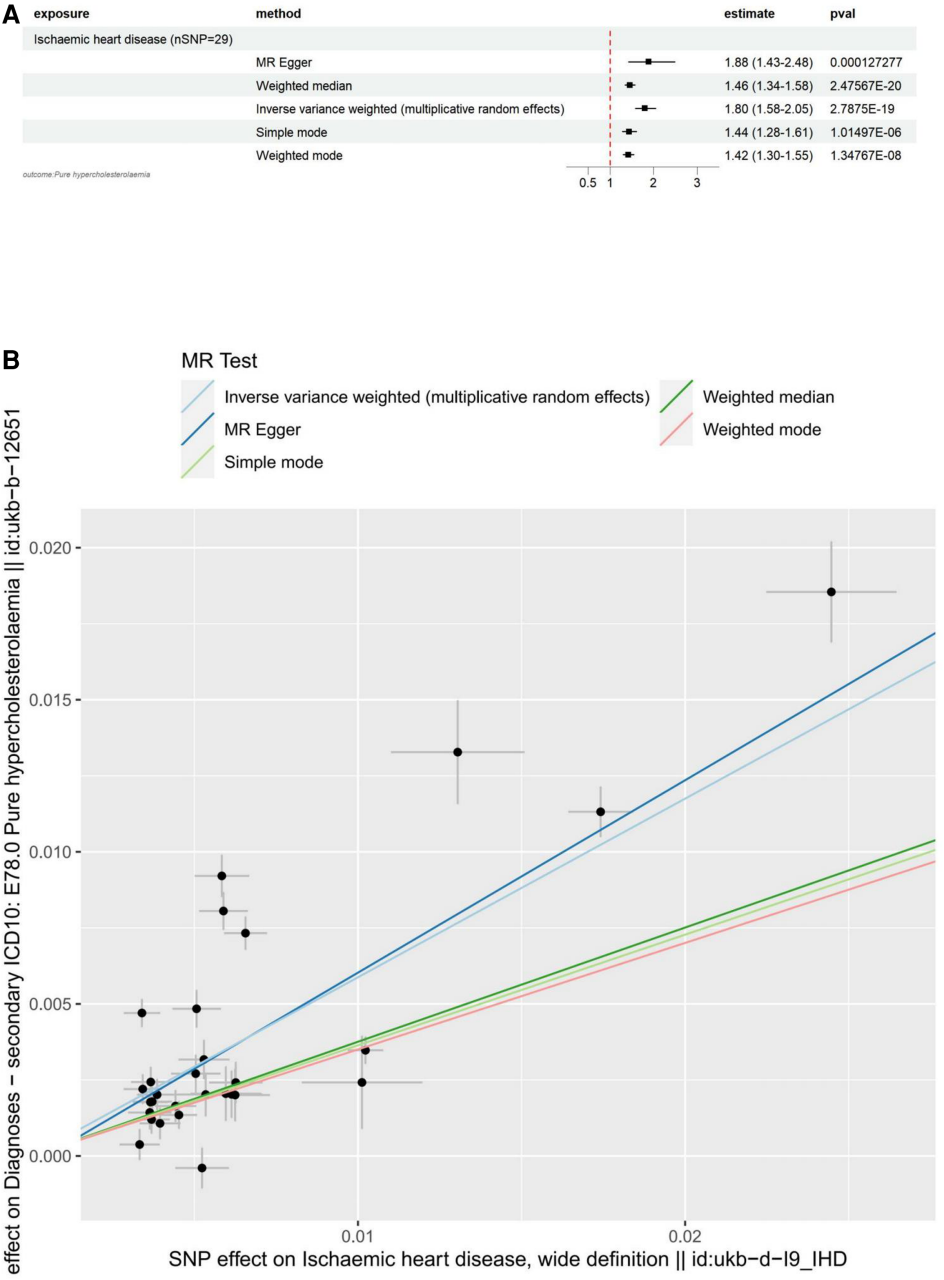
Visualization of IHD as an exposure factor in a two-sample MR. (**A**) Forest plots depicting the potential impact of SNPs associated with IHD on Hypercholesterolaemia using five MR methods. (**B**) Scatter Plots Illustrating the Effect of SNPs on IHD and its Correlation with the Effect on Hypercholesterolaemia. Circles indicate marginal genetic associations with IHD and risk of Hypercholesterolaemia for each variant. Error bars indicate 95% CIs. IHD, ischemic heart disease; MR, mendelian randomization; SNP, single nucleotide polymorphism.

To visualize these results, we utilized forest plots (Figure 3A) and scatter plots (Figure 3B). While other visualization plots used to assess heterogeneity, polymorphism and sensitivity analysis include forest plots, funnel plots, and leave-one-out plots, which we can find in Supplementary Figure S2.

### 3.2. Genetic co-localization analysis

**Figure 4:**
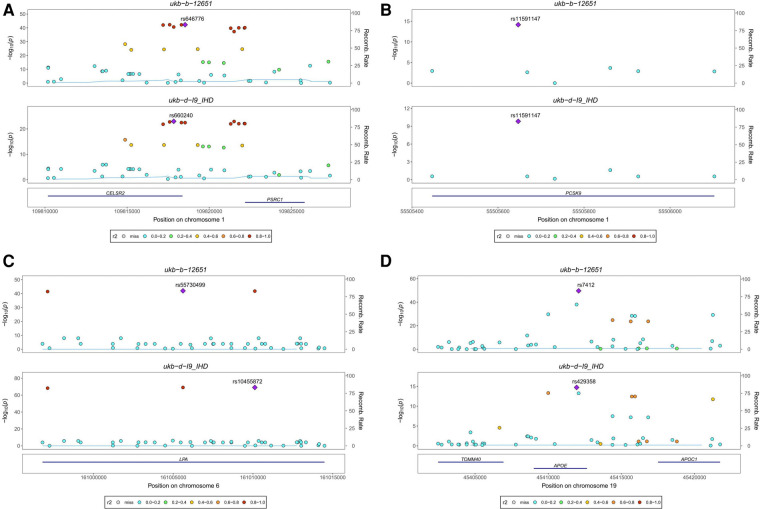
Stacked regional association plot. (**A**) CELSR2, located on chromosome 1, encodes a transmembrane protein involved in cell adhesion and lipid metabolism regulation, with SNP rs646776 associated with Pure hypercholesterolaemia and SNP rs660240 associated with IHD. (**B**) PCSK9, also located on chromosome 1, encodes a proprotein convertase that regulates LDL receptor expression. It is associated with SNP rs11591147 for both Pure hypercholesterolaemia and IHD. (**C**) LPA, located on chromosome 6, encodes lipoprotein(a) involved in cholesterol metabolism and is associated with SNP rs55730499 for Pure hypercholesterolaemia and SNP rs10455872 for IHD. (**D**) APOE, located on chromosome 19, encodes apolipoprotein E, a key player in lipid metabolism. It is associated with SNP rs7412 for Pure hypercholesterolaemia and SNP rs429358 for IHD.

Our analysis identified four colocalized genes, CELSR2, PCSK9, LPA, and APOE, that are involved in lipid metabolism and may contribute to the development of both Pure hypercholesterolaemia and IHD. CELSR2 (Figure 4A) and PCSK9 (Figure 4B) are both located on chromosome 1 and have distinct SNPs associated with each condition. LPA (Figure 4C), located on chromosome 6, and APOE (Figure 4D), located on chromosome 19, also have unique SNPs associated with each trait. CELSR2, which encodes a transmembrane protein involved in cell adhesion and lipid metabolism regulation, has SNPs rs646776 for Pure hypercholesterolaemia and rs660240 for IHD. PCSK9, encoding a proprotein convertase that regulates LDL receptor expression and is targeted by cholesterol-lowering drugs, shares the SNP rs11591147 for both conditions. LPA, encoding lipoprotein(a) and involved in cholesterol metabolism, has SNPs rs55730499 for Pure hypercholesterolaemia and rs10455872 for IHD. Lastly, APOE, encoding apolipoprotein E and playing a key role in lipid metabolism, has SNPs rs7412 for Pure hypercholesterolaemia and rs429358 for IHD. These findings provide valuable insights into the shared genetic basis of Pure hypercholesterolaemia and IHD, potentially informing the development of new therapeutic strategies.

## 4. Discussion

To shed light on the causal relationship between pure hypercholesterolemia and ischemic heart disease, we conducted bidirectional Mendelian randomization (MR) using publicly available GWAS data in both groups. Our findings reveal a bidirectional causal relationship between genetically confirmed pure hypercholesterolemia and ischemic heart disease in a European population.

First, forward MR analysis demonstrated a significant causal effect of hypercholesterolemia on IHD. The odds ratio (OR) for all five MR methods were greater than 1, signifying that hypercholesterolemia increases the risk of IHD. This finding aligns with the existing literature stating that hypercholesterolemia is a significant risk factor for cardiovascular disease (11, 21, 22). For instance, numerous studies (23–25) have shown that mononucleosis and neutrophilia, induced by hypercholesterolemia, contribute to the progression of atherosclerosis, which can subsequently lead to IHD. Additionally, Adams et al. (26) proposed in their review that oxidized LDL might trigger endothelial cell dysfunction, thereby leading to the development of ischemic syndromes. They emphasized that cellular activity could be improved within hours to days following cholesterol lowering. Importantly, another study (27) highlighted a nonlinear relationship between total cholesterol and cardiovascular disease (CVD), including IHD, with escalating mortality in IHD when total cholesterol levels exceeded 200 mg/dl. After conducting a thorough analysis, a significant association was discovered between elevated total cholesterol levels and an augmented risk of CVD mortality, specifically IHD. Our reverse MR analysis also demonstrated a significant causal effect of IHD on hypercholesterolemia. OR for all five MR methods were greater than 1, indicating that IHD increases the risk of hypercholesterolemia. This could be attributable to potential alterations in lipid metabolism resulting from the disease condition. One study (28) identified a clear association between myocardial ischemia and significant shifts in lipid metabolism. These changes were characterized by alterations in lipoprotein subclasses and an increase in total HDL cholesterol levels, especially prominent in the early post-ischemic phase.

We employed genetic co-localization analysis for hypercholesterolemia and IHD to determine whether these two phenotypes share causal genetic variants within a given region. We identified four co-localized genes CELSR2, PCSK9, LPA, and APOE. The recognition of these genes bolsters our understanding of the genetic correlation between hypercholesterolemia and IHD. Each of these genes plays a crucial role in lipid metabolism (29–32), which is a central player in the pathogenesis of both conditions. For instance, a study (29) demonstrates that CELSR2 depletion leads to a considerable reduction in lipid accumulation within hepatocytes. Additionally, it reveals that a deficiency in CELSR2 appears to undermine cell survival by inhibiting cell proliferation and encouraging apoptosis. As such, CELSR2 emerges as a potential therapeutic target for lipid metabolism regulation. PCSK9, a secreted protein primarily produced by the liver, plays a crucial role in the regulation of lipid metabolism by interacting with LDLR and other receptors, thereby contributing to cellular lipid accumulation. Its deficiency has been observed to affect heart metabolism and function, potentially leading to heart failure with preserved ejection fraction (HFpEF), indicating a strong link between PCSK9 and lipid metabolism in the cardiovascular system (33). LPA and its associated oxidized phospholipids are known to contribute significantly to chronic inflammation, a crucial factor in the development and progression of cardiovascular diseases including atherosclerosis. Further, oxidized lipids from LPA interact with immune and endothelial cells, prompting inflammatory responses that underlie various cardiovascular conditions such as myocardial infarction and calcific aortic valve stenosis, highlighting the critical role of LPA in lipid metabolism and cardiovascular health (34). Apolipoprotein E (apoE), which is predominantly synthesized in the liver, plays an integral role in lipid metabolism. This role includes mediating the clearance of triglyceride-rich lipoproteins and their remnants, initiating the reverse transport of cholesterol to the liver, and distributing lipids among cells in the nervous system. Furthermore, the various isoforms of apoE each have different impacts on the concentration of low-density lipoprotein and the risk of atherosclerosis, thereby indicating their significant influence on cardiovascular health (35). In light of these findings, the co-localised genes we identified indeed play pivotal roles in lipid metabolism, metabolic processes, and inflammatory responses, demonstrating considerable potential as therapeutic targets in hypercholesterolaemia and IHD. Specifically, PCSK9 inhibitors have emerged as a novel category of lipid-lowering agents. According to the 2019 guidelines, PCSK9 inhibitors are recommended for patients who have not attained their target lipid profile following ezetimibe/statin therapy (36). The primary mechanism of PCSK9 inhibitors involves enhancing LDL receptor density by inhibiting PCSK9 protein activity, thereby facilitating a reduction in LDL cholesterol levels (37–39). Demonstrating effectiveness in studies addressing myocardial infarction (40) and hypercholesterolaemia (39), drugs that target PCSK9 hold substantial promise for treating atherosclerosis, hypercholesterolaemia, and other related cardiovascular diseases.

In conclusion, this study provides strong evidence of causality between hypercholesterolemia and IHD and reverse causality, revealing the interaction between these two diseases that have a major impact on global health. Employing Mendelian randomization, we leveraged large-scale GWAS data on risk factors to mitigate bias from residual confounding and reverse causality, thereby minimizing the effects of population stratification (41, 42). Moreover, only cohorts predominantly of European ancestry were allowed to provide data. In accordance with the three fundamental assumptions of the MR study, our findings were robust and corroborated by various MR methods, including IVW, weighted median, and MR Egger. We also conducted an additional gene co-localisation analysis, identifying four genes, namely CELSR2, PCSK9, LPA, and APOE, co-localised with hypercholesterolaemia and IHD. Understanding these genes’ functions could aid in developing targeted therapeutic strategies for these diseases. However, our study did encounter several limitations. For example, there was significant heterogeneity across the genetic instruments in both the forward and reverse MR analyses. Although we employed the Inverse Variance Weighted method with multiplicative random effects to account for this heterogeneity, it's imperative to approach our results with caution. Another limitation stems from the genetic variants used as instruments in MR studies. Sometimes these variants can influence multiple traits or be associated with confounding factors, which can potentially bias the results. Furthermore, our findings are largely applicable to populations of European ancestry, limiting the generalizability to other ethnic groups.

## 5. Conclusions

In summary, our bidirectional MR results deliver genetic evidence, endorsing a potential causal linkage between hypercholesterolaemia and IHD. These findings also corroborate the less conventional hypothesis that IHD may instigate episodes of hypercholesterolaemia. The co-localisation analyses further intimate the presence of shared genetic causal variants, thereby presenting promising targets for the formulation of innovative therapeutic strategies.

## Data Availability

Publicly available datasets were analyzed in this study. This data can be found here: https://gwas.mrcieu.ac.uk/datasets/, using codes ukb-b-12651 and ukb-d-I9_IHD.
